# Assessment of periodontal knowledge following a mass media oral health promotion campaign: a population-based study

**DOI:** 10.1186/1472-6831-14-31

**Published:** 2014-04-05

**Authors:** Mahdia Gholami, Afsaneh Pakdaman, Ali Montazeri, Ahmad Jafari, Jorma I Virtanen

**Affiliations:** 1 Department of Community Oral Health, School of Dentistry, Tehran University of Medical Sciences, P.O. Box 1439955991 Tehran, Iran; 2 Department of Oral Public Health, Institute of Dentistry, University of Helsinki, Helsinki, Finland; 3 Mental Health Research Group, Health Metrics Research Centre, Iranian Institute for Health Sciences Research, ACECR, Tehran, Iran; 4 Department of Community Dentistry, Institute of Dentistry, University of Oulu, Oulu, Finland; 5 Oral and Maxillofacial Department, Oulu University Hospital, Oulu, Finland

**Keywords:** Periodontal disease, Mass media campaign, Oral health promotion

## Abstract

**Background:**

Oral health promotion can be achieved through education using various approaches including mass media health education campaigns. Mass media campaigns might increase oral health knowledge and perhaps could lead to desired behaviour changes and prevention of oral diseases. The aim of this study was to assess the effect of a national television campaign on knowledge of periodontal health among Iranian adults.

**Methods:**

We conducted a population-based survey among adults aged 18–50 using a stratified multistage sampling method in the 22 districts of Tehran, Iran, in 2011. All participants were interviewed at two points in time: baseline (before launching the campaign) and follow-up assessment (after the campaign was finished) by using a validated instrument. The campaign included an animation clip about periodontal health and disease that was telecasted for ten days from several national TV channels. The instrument included items related to aetiology and sign of gum disease. Periodontal knowledge score and its change were calculated for each participant and were evaluated using statistical analyses in order to examine the effect of the campaign.

**Results:**

In all 791 individuals (mean age: 32.6 years) were interviewed at baseline. Of these, 543 individuals were followed one month after the campaign. However, only 163 out of 543 reported that they had seen the campaign. Thus, comparison was made between those who had seen the campaign and who did not. The knowledge scores improved significantly among those who saw the campaign compared to those who did not (the mean knowledge score improvement 0.61 ± 0.96 versus 0.29 ± 0.8 respectively, p < 0.001). The results obtained from multiple logistic regression analysis indicated that improvement in periodontal knowledge was significantly associated with exposure to the campaign (OR = 2.20, 95% CI = 1.37-3.54), female gender (OR = 1.59, 95% CI = 1.05-2.43), being in age group 25–34 (OR = 1.76, 95% CI = 1.00-3.08), having higher education (high school: OR = 2.34, 95% CI = 1.23-4.43; university: OR = 3.33, 95% CI = 1.66-6.64), and baseline knowledge (OR = 0.25, 95% CI = 0.17-0.36).

**Conclusion:**

The study demonstrated a significant impact of the mass media campaign on Iranian adults’ knowledge regarding periodontal health and disease.

## Background

The advances in oral health research have not reached all population groups around the world and this leads to inequalities in periodontal health and other chronic diseases [1]. Periodontal diseases are highly prevalent, particularly amongst socially disadvantaged populations, impact on quality of life and are costly to treat [2]. These diseases are also associated with major chronic conditions including coronary heart disease and diabetes mellitus [3,4].

In many parts of the developing world, clinical care and chair-side prevention are both unaffordable and inappropriate for the control of periodontal diseases. The burden of disease on populations around the world could be prevented with simple and cost-effective public health interventions. There must be more focus on translating knowledge into action to improve public health [1]. The ‘common risk factor approach’, advocated by the World Health Organization (WHO), concentrates on several common causative or risk factors such as smoking and hygiene that have impact on oral diseases e.g. periodontitis and a number of important other health problems [5,6].

Oral health promotion in the form of organized activities can be used in the community level using different vehicles such as media to transfer health messages [7]. In middle and low-income countries electronic media, television and radio have been advocated as useful tools for transmission of oral health information [8,9]. Television reaches many people widespread and repeatedly [10] and can be used to transmit healthy images and messages to the public [11]. Studies conducted in different societies using media campaigns have shown that people in developing countries were more influenced by mass media interventions [12]. In fact there is evidence that mass media could improve knowledge, stimulate interests, shift in attitudes and might even change behaviours [13,14].

At present, periodontal health condition in Iran is poor. It has been reported that about 70% of young adults and 93% of middle-aged individuals were suffering from periodontal diseases as measured by the Community Periodontal Index of Treatment Needs (CPITN) [15,16]. Thus, we launched a campaign to promote oral health and prevent periodontal diseases in Iran. The campaign targeted the adults and was available throughout the country. This paper reports on the effect of a national TV campaign on knowledge of periodontal health among Iranian adults in Tehran.

## Methods

### Study design and population

This was a population-based intervention survey in Tehran, Iran. A sample of adults aged 18 to 50 selected through a stratified multi-stage area sampling. Every household within 22 districts in Tehran has the same probability of being sampled. After stratifying by district and size of residence, units (blocks) were randomly selected. Within each block, the homes were selected by random routes. All participants were interviewed at their home by a team of trained interviewers at two points in time: baseline (before launching the campaign) and follow-up (after the campaign was finished) [17]. The data were collected by the Iranian Students' Polling Agency (ISPA) [18] using a validated instrument [19].

### The campaign

A TV clip was telecasted in June 2011 through four national and one local TV channels of IRIB (Islamic Republic of Iran Broadcasting). It was broadcasted twice daily for ten days from each channel. After one week, a reminder was scheduled as a running text message, five times per day for seven days.

### Contents of the animation clip

**Figure 1:**
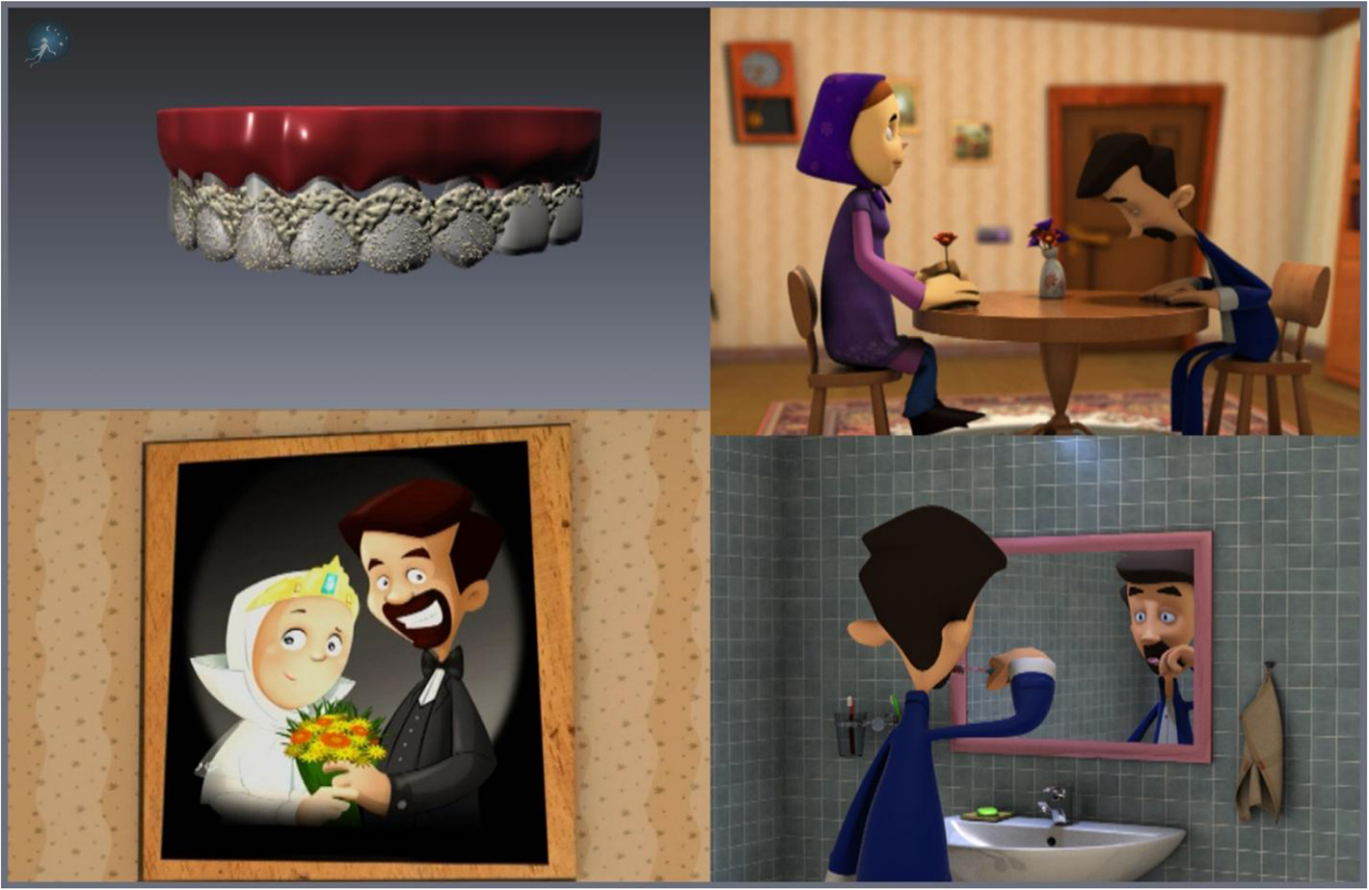
Selected pictures from the campaign.

A two-minute animation clip was developed showing a wedding ceremony in which a bridegroom is telling that I have some problems that bother me. The bride thinks that he might have convictions or perhaps this is his second marriage etc. Very soon it becomes clear that his problems are related to his gingiva including red gingiva, bleeding and mouth odour. Bride, who is a dental student, after a relaxing breath, explains to him that these problems could be solved and that dental plaque is a major reason for developing periodontal diseases. Then she explains early signs and preventive measures for the disease (Figure 1).

### The instrument

We used a valid and reliable questionnaire including items on socio-demographic background of the participants and periodontal knowledge. The background information such as age, gender, education, marital status, employment and self-reported economic status, as well as campaign visibility (seen versus not seen) were included. We used living area in square meter per person as a proxy of economic status, categorized into three levels: low <23.8 m^2^/p, moderate 23.8-36.7 m^2^/p and high ≥36.7 m^2^/p [19,20]. A three-item questionnaire derived from previously developed questionnaire (19) was used. The first question was related to the definition of dental plaque with five response categories including ‘Soft, uncolored and sticky deposits on the teeth contains microbial and food debris’, ‘Black or brown stain from food or drinks on the former teeth’, ‘It is the same as dental calculus contains microbial and food debris’, ‘Hard, colored and mineral layer on the inner surface of teeth’ and ‘Don’t know’. In the second question we asked respondents to indicate the cause of gum disease. Response categories were: ‘Oral aphtus’, ‘Dental plaque’, ‘Excessive use of antibiotics’, ‘High sugar consumption’ and ‘Don’t know’. The third question was about the early sign of gum disease with the following response categories: ‘Tooth discoloration’, ‘Tooth mobility’, ‘Tooth abrasion’, ‘Red gingival’ and ‘Don’t know’. Each item was scored on the basis of true and false responses where true was scored = 1, and false and don’t know = 0. The total score ranged from 0 to 3 with higher score indicating a better condition.

### Statistical analysis

The data were analysed for those who had both baseline and follow-up information using the SPSS package version 16 (SPSS Inc., Chicago IL, USA). Chi-square test was utilized to assess the difference between socio-demographic characteristics in two groups i.e. those who participated and those who did not participate in the follow-up. The changes of periodontal knowledge score before and after the campaign were evaluated using Wilcoxon Signed Ranks Test. In order to study the effect of independent variables such as baseline knowledge score and socio-demographic variables, logistic regression analysis was performed. For the purpose of the logistic regression analysis increase or decline in knowledge was treated as dependent variable and gender, age, education, economic status, daily time of watching TV, seeing the campaign and knowledge score at baseline were considered as independent variables.

### Ethics

The Tehran University of Medical Science Ethics Committee approved the research. Participation in the present survey was voluntary, and the data were collected anonymously. Participants gave their consent at baseline interview.

## Results

### The sample

In total, 791 individuals were interviewed at baseline. Of these, 543 responded to the questionnaire for the second time at follow-up (response rate of 68.6%) and the remaining 248 were lost to followed-up for several reasons including change in address (n =47) , dislike (n = 25) and were not available after three home visits (n =176). There were no significant differences between socio-demographic characteristics of the participants and non-participants (Table 1).

**Table 1:** Characteristics of the study participants in baseline and those who participated and lost in follow-up

	**Baseline**		**Follow-up**		**Lost to follow**		
	**N = 791**		**N = 543**		**N = 248**		
	**No. (%)**		**No. (%)**		**No. (%)**		P*
**Gender**							0.37
Male	398	(50.3)	279	(51.4)	119	(48)	
Female	393	(49.7)	264	(48.6)	129	(52)	
Total	791	(100)	543	(100)	248	(100)	
**Age group**							0.38
18-24	216	(27.3)	143	(26.3)	73	(29.4)	
25-34	257	(32.5)	178	(32.8)	79	(31.9)	
35-44	192	(24.3)	128	(23.6)	64	(25.8)	
> 45	126	(15.9)	94	(17.3)	32	(12.9)	
Total	791	(100)	543	(100)	248	(100)	
**Education**							0.62
Illiterate/Elementary^1^	126	(16.0)	91	(16.8)	35	(14.1)	
High school	336	(42.6)	229	(42.3)	107	(43.1)	
University	327	(41.4)	221	(40.9)	106	(42.8)	
Total	789	(100)	541	(100)	248	(100)	
**Marital status**							0.16
Married	276	(35.2)	183	(34.1)	93	(38)	
Single	507	(64.8)	355	(65.9)	152	(62)	
Total	783	(100)	538	(100)	245	(100)	
**Employment**							0.86
Employed	384	(48.9)	270	(50.3)	114	(46)	
Unemployed	62	(7.9)	41	(7.6)	21	(8.5)	
Student	99	(12.6)	64	(11.9)	35	(14.1)	
Housewife	224	(28.5)	152	(28.3)	72	(29)	
Other	16	(2.1)	10	(1.9)	6	(2.4)	
Total	785	(100)	537	(100)	248	(100)	
**Economic status**^**2**^							0.11
Low	227	(33.4)	164	(34.7)	63	(30.2)	
Moderate	223	(32.8)	143	(30.3)	80	(38.5)	
High	230	(33.8)	165	(35.0)	65	(31.3)	
Total	680	(100)	472	(100)	208	(100)	

*Chi-square test (p < 0.05).

^1^Illiterate/Elementary: less than nine years of education.

^2^derived from average living area in square meter per person (m^2^/p) [22].

### The campaign visibility

As indicated, only the data for 543 individuals were available at baseline and follow-up. Of these, 163 respondents indicated that have seen the campaign (30%) and 380 said that they did not (70%). Comparing the characteristics of those who seen and those who did not see the campaign indicated that there were no significant differences between these two group in gender (p = 0.10), age (p = 0.57), education (p = 0.22), marital status (p = 0.36), employment (p = 0.18) and economic status (p = 0.056).

### The campaign effect on knowledge

Overall the knowledge score increased one unit or more among individuals who had seen the campaign (52.9%) and 36.1% showed no changes in their knowledge score (Table 2). However, 11% showed one unit decrease in knowledge score. Among the respondents who did not see the campaign, the knowledge score increased one unit or more by 39.1%, had no changes by 45.5% and decreased one unit or more by 15.4%.

**Table 2:** Changes in periodontal knowledge score from baseline to follow-up among those who had seen the campaign and who did not

	**Seen**		**Not seen**	
	**No. (%)**		**No. (%)**	
**Unit change of knowledge score**				
**−2**	0.0		6	(1.6)
**−1**	17	(11.0)	51	(13.8)
**0**	56	(36.1)	168	(45.5)
**1**	58	(37.4)	121	(32.8)
**2**	18	(11.6)	20	(5.4)
**3**	6	(3.9)	3	(0.9)
**Total**	155*	(100.0)	369*	(100.0)

*Since there were some missing responses to knowledge questions, the figures do not represent 163 and 380 respectively.

**Figure 2:**
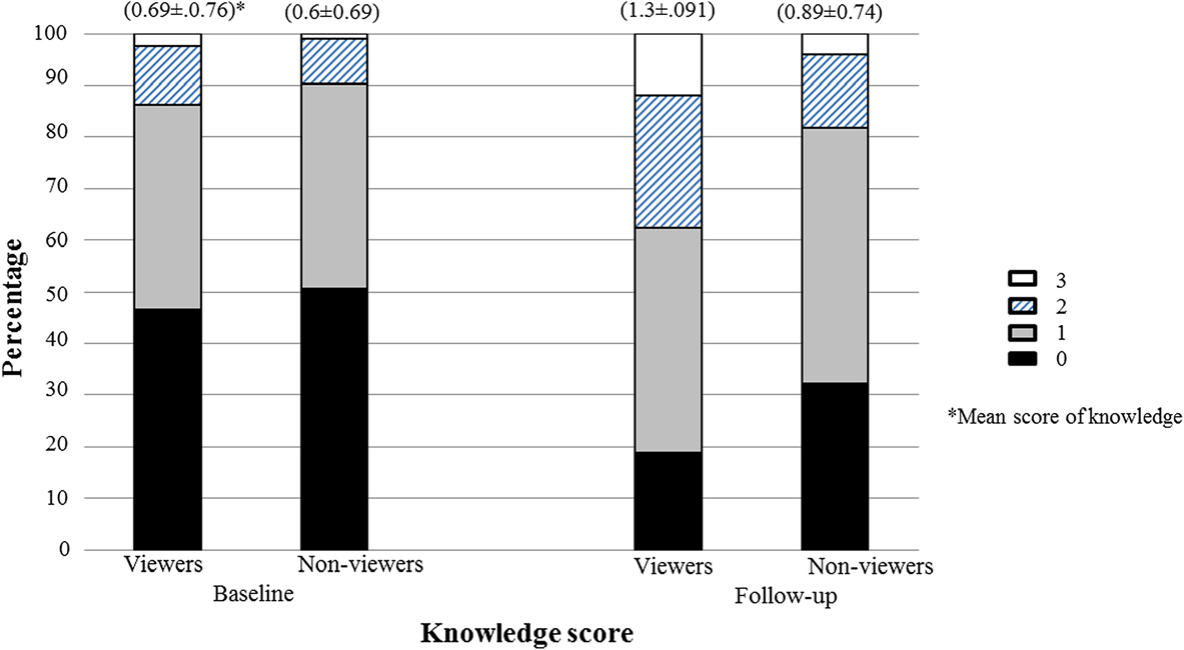
Score of knowledge (%) in baseline and follow-up among the participants who had seen the campaign and those who did not.

Knowledge score improved from baseline to follow-up and the viewers had higher scores than the non-viewers (Figure 2). The mean improvement of knowledge score was statistically significant in those who had seen the campaign (0.61 ± 0.96) compared to those who did not (0.29 ± 0.85) (p < 0.001).

Univariate analysis was performed to investigate the relationship between the independent variables and improvement of periodontal knowledge score. Female gender (OR = 1.47, 95% CI = 1.04-2.08), being in age group of 25–34 (OR = 1.68, 95% CI = 1.06-2.65), seeing the campaign (OR = 1.75, 95% CI = 1.20-2.56) and having higher baseline knowledge score (OR = 0.29, 95% CI = 0.22-0.40) were significantly related to improvement in respondents’ knowledge score. In the multivariate regression model, adjusted for all variables in the equation, the variables that significantly predicted improvement of periodontal knowledge score were seeing the campaign (OR = 2.20, 95% CI = 1.37-3.54). In addition, female gender (OR = 1.59, 95% CI = 1.05-2.43), being in age group of 25–34 (OR = 1.76, 95% CI = 1.00-3.08), having higher education (OR = 2.34, 95% CI = 1.23-4.43) (OR = 3.33, 95% CI = 1.66-6.64) and having higher baseline knowledge score (OR = 0.25, 95% CI = 0.17-0.36) were associated to the knowledge score improvement in final model (Table 3).

**Table 3:** The results obtained from logistic regression analysis for periodontal knowledge improvement

	**B**	OR* (95% CI)	**P**	**B**	OR** (95% CI)	**P**
**Gender**						
Male		1.00 (ref.)			1.00 (ref.)	
Female	0.38	1.47 (1.04-2.08)	0.028	0.46	1.59 (1.05-2.43)	0.029
**Age group**						
18-24		1.00 (ref.)			1.00 (ref.)	
25-34	0.52	1.68 (1.06-2.65)	0.025	0.56	1.76 (1.00-3.08)	0.047
35-44	0.20	1.22 (0.74- 2.01)	0.424	0.36	1.44 (0.77-2.68)	0.248
> = 45	0.41	1.51 (0.88-2.58)	0.131	0.56	1.76 (0.89-3.47)	0.104
**Education**						
Illiterate/Elementary		1.00 (ref.)			1.00 (ref.)	
High school	0.22	1.25 (0.75-2.08)	0.388	0.85	2.34 (1.23-4.43)	0.009
University	0.34	1.41 (0.84-2.35)	0.189	1.20	3.33 (1.66-6.64)	0.001
**Economic status**						
Low		1.00 (ref.)			1.00 (ref.)	
Moderate	0.02	1.02 (0.64-1.62)	0.917	-0.41	0.95 (0.56-1.61)	0.877
High	-0.20	0.81 (0.51- 1.29)	0.396	-0.17	0.84 (0.49-1.44)	0.531
**Time of watching TV**						
Rarely/Never		1.00 (ref.)			1.00 (ref.)	
One hour or less per day	0.33	1.40 (0.90-2.16)	0.130	0.50	1.65 (0.97-2.81)	0.062
Two hours or more per day	0.32	1.38 (0.80-2.35)	0.237	0.32	1.38 (0.73-2.61)	0.313
**Seeing the campaign**						
No		1.00 (ref.)			1.00 (ref.)	
Yes	0.56	1.75 (1.20-2.56)	0.004	0.79	2.20 (1.37-3.54)	0.001
**Knowledge score at baseline**	-1.20	0.29 (0.22-0.40)	<0.001	-1.36	0.25 (0.17-0.36)	<0.001

OR = odds ratio; CI = confidence intervals.

*Unadjusted or derived from univariate logistic regression analysis.

**Adjusted for gender, age, education, economic status, time of watching TV, seeing the campaign, and knowledge score at baseline.

## Discussion

This study reported on a mass media periodontal campaign in order to evaluate periodontal knowledge of Iranian adults. Overall, the campaign was well received and the study showed significant knowledge improvement among the adult population.

Similar findings have been observed previously: for instance, Mårtensson et al. showed improvement of the knowledge on periodontal disease after a mass media campaign among Swedish population [21]. Similarly, in a survey conducted in Norway, knowledge of how to prevent periodontal diseases was also increased up to three years after a mass media periodontal campaign [22]. Public awareness improvement regarding periodontal health and disease and slight improvement of oral health status has been reported following a periodontal awareness program in New Zealand [23].

Mass media has long being used an important tool for promoting public awareness in health issues. Evidence suggests that mass media campaigns can produce positive change in health knowledge, attitude and beliefs, and healthy behaviour across populations [24,25] and for several health topics including smoking cessation, HIV testing and health services utilization [26-28]. Moreover, the existing literature indicates that oral health promotion campaigns using mass media can also increase the level of knowledge, stimulate an interest, correct the attitudes and facilitate behaviour changes [10,13,14,21,29]. It has been shown that mass media intervention is effective in promoting health especially in developing countries [12]. A systematic review demonstrated a positive impact of the mass media on knowledge of HIV transmission and reduction in high-risk sexual behavior in developing countries [30]. A mass media campaign was also effective in mothers' knowledge and behavior regarding timely initiation of breastfeeding [31]. On the other hand in developed societies the effect might be weaker [32].

Females and participants aged 25–36 years showed more improvements on periodontal knowledge. These findings were in contrast to a similar study in Sweden where no statistically significant association was reported between knowledge improvement and gender or age [33]. In addition participants with higher education demonstrated much better periodontal knowledge improvement. Similar results were found by Mårtensson et al. where higher education was associated with better periodontal knowledge after the mass media campaign [33]. It seems that in designing similar campaigns the educational level of audiences is an important issue. Improvement in knowledge among those with higher education might imply that either we should choose easier messages for public or target audience based on segmentation.

We believe the findings from this study could be a good starting point in launching similar campaigns in Iran. The study used a rigorous methodology in several areas. The campaign used television for transferring oral health messages as the main route, method recently shown to be the main source of information in Iran [34]. The campaign launched through four national and one local channels and the main message was repeated hundred times in ten days. A stratified, multistage random sampling method, covering all 22 districts of Tehran, was considered to increase the representativeness of the sample. To assess periodontal knowledge we used a valid and reliable instrument created previously [19]. Data on the follow-up session was collected from the same subjects participated in the baseline interview. In order to increase responsiveness, interviewers continue data collection after the campaign to reach response rate of 70%, approximately three referrals for each subject. Additionally, dropout analysis was performed to compare characteristics of subjects who have been lost with respondents. No statistical difference was found between characteristics of the participants and non-participants in the follow-up session. At the population level this makes the dropout rate and conclusions reasonable.

The three questions may not completely capture periodontal knowledge, however, the method used was similar to studies using short questionnaire to evaluate periodontal awareness [35]. In addition the nature of our study did not allow having a control group, however the individuals who did not see the campaign were considered as controls. In fact, we compared those who seen the campaign and who did not. Huhman et al. used a similar method for comparison where they evaluated the effects of a mass media campaign on the levels of physical activity among children 9 to 13 years of age in the USA [36]. Furthermore having access to other sources of information rather than TV such as newspaper, books, etc. may influence people’s medical knowledge. Thus, one might argue that the results were contaminated with other sources of information out of our control. Although in general this is true, the interval between baseline and follow-up assessments was short and therefore it is less likely that in this short period of time any major changes have been occurred.

Health promotion campaigns through mass media are expensive and need a secure source of funding if they are to become routine. It is argued that such approaches in developing countries might work [12] particularly when those are in line with government policies. However, further evaluation of mass media oral health campaigns and their effects on behavior change is recommended.

## Conclusion

The findings from this study demonstrated a positive effect for a TV campaign on Iranian adults’ knowledge regarding periodontal health and diseases.

## Competing interests

The authors declare that they have no competing interests.

## Authors’ contributions

MG contributed to data collection, designed the study, performed the statistical analysis, and drafted the manuscript. AP designed the study, performed the statistical analyses and helped to draft the manuscript. AM contributed to study design and data analysis, and drafted the manuscript. AJ participated in the design of the study and helped to draft the manuscript. JV contributed to study design and data analysis, and helped to draft the manuscript. All authors read and approved the final manuscript.

## Pre-publication history

The pre-publication history for this paper can be accessed here:

http://www.biomedcentral.com/1472-6831/14/31/prepub
